# Metagenomic sequencing sheds light on microbes putatively associated with pneumonia-related fatalities of white-tailed deer (Odocoileus virginianus)

**DOI:** 10.1099/mgen.0.001214

**Published:** 2024-03-27

**Authors:** Melanie B. Prentice, Marie L. J. Gilbertson, Daniel J. Storm, Wendy C. Turner, Daniel P. Walsh, Marie E. Pinkerton, Pauline L. Kamath

**Affiliations:** 1 School of Food and Agriculture, University of Maine, Maine, USA; 2 Wisconsin Cooperative Wildlife Research Unit, Department of Forest and Wildlife Ecology, University of Wisconsin-Madison, Wisconsin, USA; 3 Wisconsin Department of Natural Resources, Wisconsin, USA; 4 U.S. Geological Survey, Wisconsin Cooperative Wildlife Research Unit, Department of Forest and Wildlife Ecology, University of Wisconsin-Madison, Wisconsin, USA; 5 U.S. Geological Survey, Montana Cooperative Wildlife Research Unit, University of Montana, Montana, USA; 6 Department of Pathobiological Sciences, School of Veterinary Medicine, University of Wisconsin-Madison, Wisconsin, USA; 7 Maine Center for Genetics in the Environment, University of Maine, Orono, Maine, USA

**Keywords:** epidemiology, metagenomics, pneumonia, white-tailed deer, wildlife disease

## Abstract

With emerging infectious disease outbreaks in human, domestic and wild animal populations on the rise, improvements in pathogen characterization and surveillance are paramount for the protection of human and animal health, as well as the conservation of ecologically and economically important wildlife. Genomics offers a range of suitable tools to meet these goals, with metagenomic sequencing facilitating the characterization of whole microbial communities associated with emerging and endemic disease outbreaks. Here, we use metagenomic sequencing in a case-control study to identify microbes in lung tissue associated with newly observed pneumonia-related fatalities in 34 white-tailed deer (*Odocoileus virginianus*) in Wisconsin, USA. We identified 20 bacterial species that occurred in more than a single individual. Of these, only *Clostridium novyi* was found to substantially differ (in number of detections) between case and control sample groups; however, this difference was not statistically significant. We also detected several bacterial species associated with pneumonia and/or other diseases in ruminants (*Mycoplasma ovipneumoniae*, *Trueperella pyogenes*, *Pasteurella multocida*, *Anaplasma phagocytophilum*, *Fusobacterium necrophorum*); however, these species did not substantially differ between case and control sample groups. On average, we detected a larger number of bacterial species in case samples than controls, supporting the potential role of polymicrobial infections in this system. Importantly, we did not detect DNA of viruses or fungi, suggesting that they are not significantly associated with pneumonia in this system. Together, these results highlight the utility of metagenomic sequencing for identifying disease-associated microbes. This preliminary list of microbes will help inform future research on pneumonia-associated fatalities of white-tailed deer.

**Impact Statement**

This research highlights the utility of metagenomic sequencing for characterizing microbes associated with novel disease outbreaks when the pathogen responsible for morbidity and/or mortality is unknown. The benefit of metagenomic sequencing over traditional pathogen characterization techniques is its ability to characterize the complete suite of bacterial, viral and/or fungal pathogens present within a sample without prior knowledge of target pathogens. Given this, metagenomic sequencing is especially useful for studying diseases that are polymicrobial (i.e. involve more than a single pathogen, especially if non-bacterial agents are involved). Most frequently applied in studies of human diseases, metagenomic sequencing is an underused tool in wildlife disease research due to challenges with sample collection and the cost of sample processing. However, given the potential exchange of pathogens between wildlife, domestic animals and/or human hosts, improved methods of detecting, monitoring, and managing disease outbreaks in wildlife will ultimately benefit human and domestic animal health, food security, and conservation. Here, we demonstrate the use of metagenomic sequencing to identify the microbes associated with recently observed pneumonia-related fatalities in white-tailed deer. Characterizing potential pathogens associated with these fatalities is the first step towards understanding the distribution and severity of this disease in deer, as well as its impacts on deer population health.

## Data Summary

Metagenomic sequencing datasets generated in this study are publicly available in the NCBI SRA data repository (BioProject PRJNA1025254, Accession No. SAMN37717265-SAMN37717298). All sample metadata are provided within the manuscript. The script generated for bioinformatic analyses is provided as supplementary material.

## Introduction

Outbreaks of wildlife diseases over the last several decades have affected a wide range of species, with devastating consequences. In wildlife, emerging disease can lead to the substantial decline or complete extirpation of populations, e.g. avian influenza in birds [1], chytridiomycosis in amphibians [2], chronic wasting disease in cervids [3], white-nose syndrome in bats [4] and wasting disease in sea stars [5] and seagrasses [6]. The impacts of such outbreaks may also extend to human and/or domestic animal health via spillover events. Indeed, estimates suggest that zoonotic diseases account for 60 % of emerging infectious diseases, 72 % of which originate from wildlife hosts and are significantly increasing with time [7]. Collectively, the emergence of novel pathogens across wild taxa and ecosystems calls for improved research methods and tools for detecting, monitoring, and managing disease outbreaks in often rapid and quickly evolving timeframes.

In line with our need for rapid solutions to combat emerging infectious disease outbreaks, the growing field of genomics is producing novel tools and methodologies for disease surveillance [8,9], pathogen strain typing [10,11] and vaccine development [12,13]. In particular, the development of genomic tools that facilitate the reliable characterization and surveillance of pathogens has overcome many of the limitations of more conventional surveillance methods. For example, microbiological culturing methods often rely on *a priori* knowledge of the pathogen responsible, preventing their application to emerging infectious disease outbreaks when etiological agents are unknown. Further, in cases where the pathogen of interest has been characterized, surveillance can be difficult for organisms that are not easily cultured (e.g. viruses) and is further complicated by complex polymicrobial infections. In contrast, genomic techniques can be deployed to either identify suspected pathogens of interest via targeted amplicon sequencing, or to characterize the entire microbial community. The latter, termed ‘clinical metagenomics,’ offers the ability to characterize the complete suite of bacterial, viral and/or fungal pathogens present within a sample, thus facilitating both pathogen discovery and surveillance in a single experiment [8].

In clinical settings, metagenomic approaches have been successfully employed to rapidly identify pathogens associated with human morbidity and mortality (e.g. [14]). In contrast, the application of genomic tools to wildlife diseases is relatively underrepresented [15,16]. Specifically, the collection of wildlife disease data is challenged by regulatory issues, financial burden, inconsistent or incomplete collection of samples and associated metadata, time and safety constraints when dealing with challenging terrain and putative zoonotic diseases, the often-suboptimal condition of field-collected samples from rapidly decomposing carcasses, and poor sample preservation [17]. Sample collection is especially difficult for large carcasses which must be sampled on site, requiring training in necropsy techniques and biosafety. Combined, these challenges limit the number of suitable wildlife samples available for study. Post collection, additional challenges are encountered due to the added costs of genomic work and the expertise required to bioinformatically analyse and interpret the data [16,18]. However, with continued development and cost reductions of genomic sequencing technologies (e.g. [19] and applications thereof, such as [20]) alleviating some of the aforementioned challenges, genomic research is poised to rapidly expand our understanding of, and thus ability to respond to, wildlife disease outbreaks for the protection of ecologically and economically important species or populations thereof.

Cervids are a central component of forested ecosystems across North America, with significant ecological, cultural, and economic value. In 2016, recreational hunting of deer (*Odocoileus* sp.) accounted for $20.9 billion of the United States Gross Domestic Product, with an additional $3.1 billion and $1.9 billion earned from federal and state tax revenues, respectively [21]. Importantly, cervids also serve as hosts for many pathogens originating in domestic animals, humans and wildlife reservoirs, including those that cause chronic wasting disease (CWD, [3]), bovine tuberculosis [22], brucellosis [23], and most recently, COVID-19 [24]. Not only do many of these diseases have substantial impacts on the health and persistence of cervid populations, but the relatively close evolutionary relationships between cervids and some domestic species (primarily Bovidae) suggests that the effects of outbreaks in these populations may have additional consequences via spillover or spillback to livestock (e.g. [25,26]) and humans (e.g. [27]).

In addition to the diseases already circulating in cervid populations, novel emerging pathogens present another threat. In south-central Wisconsin, USA, white-tailed deer (*Odocoileus virginianus*) populations have been extensively monitored since 2001 due to the impacts of CWD on deer populations in the region [28]. In 2017, collaring of deer identified morbidity and mortality in white-tailed deer that were not infected with CWD, and have since been attributed to pneumonia [29]. Interestingly, pneumonia was not significantly associated with nutritional condition, and individuals with good body condition were often found with severe lesions [29].

Pneumonia is a complex respiratory disease involving inflammation of lung tissue that can be caused by viral, bacterial, or fungal pathogens, but may also be polymicrobial [30]. Diagnosis of pneumonia in deer is accomplished by observation of inflammatory lesions in the lung tissue. Having been observed in many wildlife systems (e.g. [31,34]), pneumonia is perhaps most significantly attributed to the historical declines and regional extirpations of bighorn sheep (*Ovis canadensis*) populations throughout North America [35]. While the complex aetiology of pneumonia in bighorn sheep is still unresolved, the bacterium *Mycoplasma ovipneumoniae* is thought to play a central role in the initiation of outbreaks, following contact with domestic sheep and goats, which are known carriers of *M. ovipneumoniae* [36,37]. Once introduced, female carriers can transmit infections to susceptible lambs, limiting recruitment and preventing population recovery [38]. The absence of traditional treatments such as vaccines for pneumonia, and the complex aetiology of the disease has historically limited the success of management programmes, and once exposed, bighorn sheep populations continue to experience high morbidity and mortality attributed to pneumonia [36]. Considering this, the observation of pneumonia in other wildlife species is cause for concern and warrants timely investigation to inform the management of impacted species and populations.

To date, pneumonia-related morbidity and mortality has been reported in a total of 86 white-tailed deer in Wisconsin (51.2 % of evaluated individuals), ~60 % of which have been classified as moderate to severe in grade [29]. While it may not represent the ultimate cause of death in all individuals, and some level of pneumonia is expected due to the high prevalence of CWD in this area, the observation of a high rate of severe pneumonia lesions cannot be fully explained by the presence of CWD alone [29]. Currently, the aetiologic agent for pneumonia in this population remains unknown as culturing methods have been unable to definitively identify a candidate [29]. Given these considerations, metagenomic sequencing offers an alternative method for pathogen identification in this system. Here, we present the results of a pilot study using long-read metagenomic sequencing in a case-control study design to identify the microbial communities associated with pneumonia-related fatalities of white-tailed deer in the state of Wisconsin. Ultimately, identification of the agent(s) responsible for these fatalities will not only enable its surveillance but will also facilitate future research on the population-level impacts of the disease, and its interaction with other circulating pathogens in this area, including the prion causing CWD.

## Methods

### Study area & sampling

In 2017–2020, 1065 white-tailed deer were collared by the Wisconsin Department of Natural Resources (WDNR) personnel in south-central Wisconsin for CWD monitoring and research. This area represents the core of the CWD distribution in the state, encompassing the area where CWD was first detected in Wisconsin. At the time of capture, WDNR staff recorded age, sex, and standard body measurements of individual deer, which were subsequently collared and monitored [29]. Typically, within 24 h of receiving a mortality signal, approximately one-third of deceased deer (141/433 mortalities evaluated) were collected by WDNR staff and taken to the Wisconsin Veterinary Diagnostic Laboratory for necropsy [29]. Deer capture and handling protocols were approved under Wisconsin Department of Natural Resource’s Animal Care and Use Committee (Protocol 16-Storm-01).

### Necropsy & diagnosis

Nutritional condition and cause of death was diagnosed during laboratory necropsy as described in Gilbertson *et al*. [29]. Gross pneumonia lesions were detected and described by a board-certified veterinary pathologist and evaluated histologically. Identification of pneumonia aetiologic agents was attempted using aerobic culture, but agents were typically not able to be isolated and definitively identified (Table 1).

**Table 1.** Metadata for 34 samples used in a study of white-tailed deer (*Odocoileus virginianus*) pneumonia in Wisconsin, USA. Sex is classified as male (M) or female (F). Cause of death is defined by broad groupings, where infectious causes of death are further subdivided into pneumonia-related, non-pnemonia-related and mixed infections. “Other” causes of death include myocardial necrosis and cerebellar herniation. No data is indicated by “--”

Sample	Sex	Age class	Case/ Control	Body condition	Cause of death	Pneumonia class	Pneumonia grade	Culture/Histology Results
18NX35	M	<1	Control	Poor	Trauma	--	--	--
18NX37	F	2–4	Control	Fair	Predation	--	--	--
18NX41	F	1–2	Control	Poor	Predation	--	--	--
19NX11	F	2–4	Control	Good	Trauma	--	--	--
19NX12	M	<1	Control	Good	Trauma	--	--	--
19NX88	M	2–4	Control	Good	Infectious†	--	--	--
19NX09	F	<1	Control	Fair	Infectious†, Other	--	--	--
19NX10	M	1–2	Control	Poor	Infectious†	--	--	--
19NX15	F	<1	Control	Fair	Infectious†, Other	--	--	--
19NX23	M	<1	Control	Poor	Predation	--	--	--
19NX25	M	<1	Control	Poor	Harvest	--	--	--
19NX27	M	<1	Control	Severe-Poor	Nutritional	--	--	--
19NX28	F	<1	Control	Poor	Predation	--	--	--
19NX42	F	≥8	Control	Poor	Other	--	--	--
19NX43	F	2–4	Control	Poor	Infectious†	--	--	--
19NX75	F	<1	Control	Poor-Fair	Infectious†	--	--	--
20NX01	F	1–2	Control	Poor	Trauma	--	--	--
17NX48	F	2–4	Case	Good	Infectious‡	Pleuropneumonia	Severe	Nonspecific bacteria
18NX24	M	2–4	Case	Fair	Infectious‡	Pleuropneumonia	Severe	*Trueperella pyogenes*, *Bibersteinia trehalosi*
18NX44	F	<1	Case	Poor	Infectious*	Bronchopneumonia	Mixed Severity	*Trueperella pyogenes*, Nonspecific bacteria
18NX66	M	1–2	Case	Excellent	Infectious‡	Pneumonia	Severe	Nonspecific bacteria
19NX41	M	4–6	Case	Poor	Infectious*	Bronchopneumonia	Severe	*Trueperella pyogenes*, *Pasteurella multocida*, *Escherichia coli*
19NX80	M	2–4	Case	Good	Infectious*	Bronchopneumonia	Severe	Nonspecific bacteria
19NX90	M	<1	Case	Good	Infectious‡	Bronchopneumonia	Moderate	Nonspecific bacteria
18NX29	M	1–2	Case	Fair	Trauma	Bronchopneumonia	Mild	None
18NX36	M	2–4	Case	Good	Trauma	Pleuropneumonia	Mild	Nonspecific nematode
18NX43	M	1–2	Case	Good	Trauma	Pneumonia	Mild	None
18NX63	M	<1	Case	Poor	Infectious*	Bronchopneumonia	Moderate-Severe	None
19NX07	F	≥8	Case	Excellent	Infectious‡	Interstitial pneumonia	Mild	Nonspecific bacteria
19NX36	M	<1	Case	Poor	Predation	Bronchopneumonia	Severe	None
19NX64	M	<1	Case	Poor	Infectious‡	Interstitial pneumonia	Mild	Nonspecific bacteria
20NX07	F	<1	Case	Good	Infectious*	Pneumonia	Mild	Nonspecific nematode
20NX09	F	2–4	Case	Fair	Infectious‡	Pneumonia	n/a	Nonspecific bacteria
21NX08	M	2–4	Case	Severe-Poor	Infectious*	Pneumonia	Severe	*Trueperella pyogenes*

*Pneumonia-related infection.

†Non-pneumonia-related infection.

‡Mixed infection.

CWD testing of individuals was conducted via immunohistochemistry on lymphoid or brain tissue samples taken from carcasses [29]. Lung tissue samples, including tissue from gross lung lesions, were also collected at this time and stored at −80 °C until the time of DNA extraction.

### DNA extraction & metagenomic sequencing

A subset of lung tissue samples taken during necropsies were selected for metagenomic sequencing (Table 1). We used a case-control study design, comparing individuals for which significant pneumonia was observed at death (‘case samples,’ *N*=17) to those which died during the same time period due to non-respiratory causes of death (‘control samples,’ *N*=17). To exclude potential interactions of the lung microbial community and CWD, we eliminated all individuals that tested positive for CWD from this study; two individuals lacked CWD testing results but being less than one year of age at death, were unlikely to be clinically affected by CWD even if infected. We also avoided cases of interstitial pneumonia (inflammation affecting the interstitial tissue between the air sacs of the lungs), focusing instead on bronchopneumonia (inflammation originating in the airways [bronchi] of the lungs) or mixed-pneumonia, to exclude the possibility of pneumonia resulting from infections that started outside of the lungs (Table 1). To assess the possible effects of age, sex, and body condition, we tested for significant differences in the distribution of these variables between case and control sample groups using chi-squared tests in R (v4.1.3 [39]) with Yate’s continuity correction to account for small sample sizes.

Genomic DNA was extracted from lung tissue using a Qiagen DNeasy PowerLyzer PowerSoil Kit (Qiagen, Hilden, Germany) according to the manufacturer’s protocol with minor modifications. Specifically, for the homogenization of lung tissue, we used two intervals of 3500 r.p.m. for 45 s separated by a 30 s interval, and subsequently centrifuged bead tubes at 10 000 ***g*** for 90 s. We quantified DNA using the dsDNA Broad Range Assay kit on a Qubit 4 Fluorometer (Invitrogen, Waltham, MA, USA) and assessed DNA quality using 1 % agarose gels. RNA was not extracted for this project as field samples were not preserved to maintain RNA.

Genomic sequencing was conducted on a MinION long-read sequencing platform (Oxford Nanopore Technologies, Oxford, UK [40]). Library preparations were performed using the SQK-LSK 109 ligation sequencing kit (Oxford Nanopore Technologies, Oxford, UK) following the manufacturer’s protocol. While MinION sequencing allows high multiplexing capabilities, we chose to multiplex samples in groups of three to maintain high sequence read output for each sample, thus increasing the probability that we detect rare microbial species present at lower abundance within our samples. Multiplexing samples in groups of three meant that a single leftover sample was sequenced on its own flow cell. Sequencing was conducted on R9.4.1 flow cells, which can generate up to 50 Gb of data (Oxford Nanopore Technologies, Oxford, UK). Each run was performed over 72 h.

### Bioinformatics & data analysis

Raw data were base-called and de-multiplexed using Guppy v6.0.0 (Oxford Nanopore Technologies Oxford, UK). Quality control of sequencing runs and individual samples was performed using MinIONQC [41] and FastQC v0.11.7 [42], respectively. FastQC results were summarized across samples using MultiQC v1.10.1 [43]. Read data were aligned to the draft white-tailed deer genome (RefSeq GCF_002102435.1) using BWA-MEM v0.7.17 [44]. Following alignment, reads aligning to the white-tailed deer genome were discarded, retaining unmapped reads (i.e. those that did not map to the host genome) which were filtered to remove reads shorter than 200 base pairs using a custom script. The resulting sequence dataset for each sample was queried against the NCBI nucleotide database using blastn v2.11.0 (RRID:SCR_001653) with an e-value cut-off of 10^−10^ and a minimum percent identity threshold of 90 %.

For each query sequence (i.e. each read in an individual sample’s dataset), we selected the top ten blast results from which we removed duplicate results and results produced from alignments shorter than 100 bp in length. Results were then summarized across all query sequences to produce a list of the species identified and the number of times each species was detected in each sample. From these lists, we removed any non-microbial species (e.g. mammal species which may represent poor alignment of some host DNA sequences to the draft genome). To account for the potential for false positive results, we also removed any species that were identified by fewer than 100 sequences. Our final lists contained only microbial species reliably identified (i.e. by >100 sequences) in each sample. From this list, we removed any microbial species that were not identified at the species level (i.e. genus and family-level characterizations), as these results were considered too nonspecific to interpret. Combining results across samples, we further reduced our list of microbes of interest by removing any species that were detected in only a single individual. This allowed us to produce a concise list of species that were less likely to represent sequencing or taxonomic classification errors that could be used for statistical comparison of case and control groups. We then calculated the proportion of case samples and the proportion of control samples for which each species was detected. We examined whether these proportions differed significantly between the case versus control groups using chi-squared tests in R, with Yate’s continuity correction for small sample sizes. These results were used to identify putative pathogens associated with pneumonia-related mortality in deer (*P*<0.05).

Given that one sample was sequenced on a flow cell by itself, the amount of data produced for this sample was significantly greater than that of all other samples. To assess the potential impact that this increase in sequencing power had on our results, we randomly subset the sequencing data for this sample to the average number of reads generated across all other samples and re-ran the bioinformatic analysis described above. Comparison of the two datasets suggests that while some species with low frequency detections (<300 sequences) in the full dataset were no longer detected in the data subset, all microbe species with relevance to the results presented here (i.e. those found in >1 individual) were consistently detected in both sets of data. Thus, we present the results from the data subset here for the sake of consistency.

## Results

Metagenomic sequencing resulted in datasets containing between 1.5–5.7 million reads (mean=1.7 million) per sample, with a per-sample average read length ranging from 600 to 8367 bp (overall mean=2678 bp) across samples (Table 2). Sequence quality was typical for long-read sequencing, with average Q-values ranging between 11–13 (mean=12; Table S1, available in the online version of this article). Unmapped reads (i.e. those that did not align to the host genome, and thus potentially correspond to microbial sequences) comprised between 0.26–5.59 % (mean=0.84 %; 16 872 reads) of each sample’s dataset, of which between 4.07–77.72 % (mean=43.85 %; 6913 reads) produced a match with the NCBI nucleotide database using blast (Table 2).

**Table 2.** Summary of sequencing statistics for samples used in a study of white-tailed deer (*Odocoileus virginianus*) pneumonia in Wisconsin, USA. Unmapped reads represent reads that did not align to the white-tailed deer reference genome and were thus of interest to this study (i.e. microbe sequences), filtered to remove sequences shorter than 200 bp (base pairs). We also present the number (and percentage, in parentheses) of reads for which we did and did not identify a sequence match to the National Centre for Biotechnology Information (NCBI) nucleotide database. The last column contains a count of the number of different microbe species (with >100 read matches) identified in each sample dataset. Summary statistics across all samples are presented at the bottom of the table; for the number of microbe species identified, we present summary statistics separately for case and control samples (case / control)

Sample	Case/control	Total no. of reads	Average % GC content	Average sequence length (bp)	No. (percent) of unmapped reads (>200 bp)	No. (percent) of unmapped reads with blast results (>200 bp)	No. (percent) of unmapped reads without blast results (>200 bp)	# Putative pathogen species identified
17NX48	Case	1 429 374	43	2048	16 413 (1.15)	12 756 (77.72)	3657 (22.28)	1
18NX24	Case	2 087 318	43	1090	12 912 (0.62)	7370 (57.08)	5542 (42.92)	7
18NX29	Case	1 794 457	42	1937	7667 (0.43)	3169 (41.33)	4498 (58.67)	1
18NX36	Case	1 505 078	43	5666	7453 (0.50)	3211 (43.08)	4242 (56.92)	8
18NX43	Case	566 047	43	2167	3559 (0.63)	1264 (35.52)	2295 (64.48)	2
18NX44	Case	1 239 301	43	3077	10 603 (0.86)	7923 (74.72)	2680 (25.28)	2
18NX63	Case	1 252 963	43	2415	6246 (0.50)	2257 (36.14)	3989 (63.86)	1
18NX66	Case	1 216 762	44	1242	8457 (0.70)	4283 (50.64)	4174 (49.36)	4
19NX07	Case	2 440 768	43	1308	17 908 (0.73)	10 316 (57.61)	7592 (42.39)	3
19NX36	Case	1 653 219	43	2020	8501 (0.51)	3745 (44.05)	4756 (55.95)	3
19NX41	Case	1 274 729	42	2121	15 641 (0.85)	6373 (40.75)	9268 (59.25)	9
19NX64	Case	5 716 300	41	1063	36 725 (0.64)	16 897 (46.01)	19 828 (53.99)	1
19NX80	Case	1 700 000	43	1006	17 977 (1.06)	12 911 (71.82)	5066 (28.18)	7
19NX90	Case	4 739 028	43	600	40 464 (0.85)	19 446 (48.06)	21 018 (51.94)	7
20NX07	Case	1 024 947	43	1829	5280 (0.52)	2202 (41.70)	3078 (58.30)	2
20NX09	Case	636 717	43	2359	3066 (0.48)	1202 (39.20)	1864 (60.80)	1
21NX08	Case	737 343	43	2198	2778 (0.38)	1389 (50.00)	1389 (50.00)	1
18NX35	Control	323 395	43	8367	2110 (0.65)	325 (15.40)	1785 (84.60)	0
18NX37	Control	2 931 756	44	1003	121 452 (4.14)	70 219 (57.82)	51 233 (42.18)	6
18NX41	Control	1 846 451	42	2020	7964 (0.62)	2970 (37.29)	4994 (62.71)	1
19NX09	Control	3 041 854	43	1366	15 298 (0.50)	5815 (38.01)	9483 (61.99)	1
19NX10	Control	713 602	42	3710	3647 (0.51)	1456 (39.92)	2191 (60.08)	4
19NX11	Control	1 512 907	43	1220	7665 (0.51)	3397 (44.32)	4268 (55.68)	0
19NX12	Control	2 675 380	43	1434	10 200 (0.38)	4866 (47.71)	5334 (52.29)	0
19NX15	Control	541 845	43	4257	1393 (0.26)	598 (42.93)	795 (57.07)	0
19NX23	Control	2 386 130	43	2091	25 715 (1.08)	3943 (15.33)	21 772 (84.67)	0
19NX25	Control	200 640	43	4104	913 (0.46)	434 (47.54)	479 (52.46)	2
19NX27	Control	153 588	43	8251	602 (0.39)	172 (28.57)	430 (71.43)	1
19NX28	Control	310 677	43	8169	838 (0.27)	375 (44.74)	463 (55.25)	1
19NX42	Control	1 170 963	43	2319	5010 (0.43)	2518 (50.26)	2492 (49.74)	2
19NX43	Control	1 987 837	44	1146	111 114 (5.59)	4524 (4.07)	106 590 (95.93)	2
19NX75	Control	1 790 698	43	3032	9042 (0.50)	3289 (36.37)	5753 (63.63)	3
19NX88	Control	4 304 397	43	1214	25 771 (0.60)	12 178 (47.25)	13 593 (52.75)	6
20NX01	Control	1 008 613	43	3215	3267 (0.32)	1244 (38.08)	2023 (61.92)	2
	**MINIMUM**	153 588	41	600	602 (0.26)	172 (4.07)	430 (22.28)	1/0
	**MEDIAN**	1 467 226	43	2070	8221 (0.51)	3343 (43.57)	4255 (56.43)	2/1
	**MEAN**	1 703 385	43	2678	16 872 (0.84)	6913 (43.85)	9959 (56.15)	3.5/1.8
	**MAXIMUM**	5 716 300	44	8367	121 452 (5.59)	70 219 (77.72)	106 590 (95.93)	9/6

Across all samples, a total of 75 microbial species were identified by at least 100 reads in at least one sample (Table S2). The majority of these species were bacterial, with five phages identified in a single individual each. Of those 75 species, 20 bacterial species were found in more than a single individual, and 13 in more than two individuals (Table 3). Of the 20 species, only *Clostridium novyi* was found to substantially differ (in the number of detections) between case and control sample groups; however, this difference was not statistically significant (X-squared=3.5417, df=1, *P*=0.06). Although not significant, we also detected several bacterial species that have been associated with pneumonia and/or other diseases of ruminants, including *Mycoplasma ovipneumoniae* (also known as *Mesmoycoplasma ovipneumoniae*), *Anaplasma phagocytophilum*, *Trueperella pyogenes*, *Pasteurella multocida*, and *Fusobacterium necrophorum*. Both *T. pyogenes* and *P. multocida* were also identified via culturing, with varying levels of agreement between the culturing and metagenomic datasets (4/5 and 1/5 individuals with metagenomic sequence detections also had positive culture results for *T. pyogenes* and *P. multocida*, respectively; Table S3). Interestingly, we detected a larger number of bacterial species in our case samples compared to our control samples; on average, case samples contained 3.5 different species, whereas control samples contained 1.8 (Table 2). We did not detect fungi or DNA viruses of eukaryotes in any of our samples.

**Table 3.** List of microbes identified in lung tissue samples from 34 white-tailed deer (*Odocoileus virginianus*) mortalities from Wisconsin, USA.This list contains species identified by at least 100 reads in >1 sample. Sample total represents the total number of samples (out of *N*=34 total) from which each species was identified, with subsequent columns showing results for the number of detections in case (*N*=17) and control (*N*=17) samples. Species names are reported based on the name attributed to the blast match of the National Centre for Biotechnology Information (NCBI) nucleotide database. *P*-values are from chi-squared tests comparing the number of occurrences of each species between case and control sample groups. Species in bold represent those that differed with a *P*-value <0.1

Species	Sample total	Case sample total (percentage)	Control sample total (percentage)	*P*-value
*Anaplasma phagocytophilum*	3	3 (17.65)	0 (0.00)	0.2266
*Bacteroides fragilis*	3	1 (5.88)	2 (11.76)	1
*Clostridium botulinum*	13	9 (52.94)	4 (23.53)	0.1581
***Clostridium novyi***	**10**	**8** (47.06)	**2** (11.76)	**0.0599**
*Clostridium perfringens*	2	1 (5.88)	1 (5.88)	1
*Clostridium septicum*	2	2 (11.76)	0 (0.00)	0.4661
Clostridium sordellii*	3	3 (17.65)	0 (0.00)	0.2266
*Escherichia coli*	9	3 (17.65)	6 (35.29)	0.4369
*Fusobacterium gonidiaformans*	4	3 (17.65)	1 (5.88)	0.5945
*Fusobacterium necrophorum*	4	3 (17.65)	1 (5.88)	0.5945
*Fusobacterium nucleatum*	3	2 (11.76)	1 (5.88)	1
*Mycoplasma ovipneumoniae†*	4	3 (17.65)	1 (5.88)	0.5945
Paeniclostridium sordellii*	4	3 (17.65)	1 (5.88)	0.5945
*Pasteurella multocida*	10	5 (29.41)	5 (29.41)	1
*Pseudomonas fragi*	2	0 (0.00)	2 (11.76)	0.4661
*Streptococcus equinus*	2	2 (11.76)	0 (0.00)	0.4661
*Streptococcus infantarius*	2	2 (11.76)	0 (0.00)	0.4661
*Streptococcus lutetiensis*	2	2 (11.76)	0 (0.00)	0.4661
*Streptococcus ruminantium*	2	0 (0.00)	2 (11.76)	0.4661
*Trueperella pyogenes*	7	5 (29.41)	2 (11.76)	0.3963

* These results represent sequence matches of the same species under different names. Combining their results (6six case detections, 1one control detections), is not significant (X-squared=2.878, df=1, *P*=0.09).

† Also known as *Mesomycoplasma ovipneumoniae.*

**Fig. 1.**
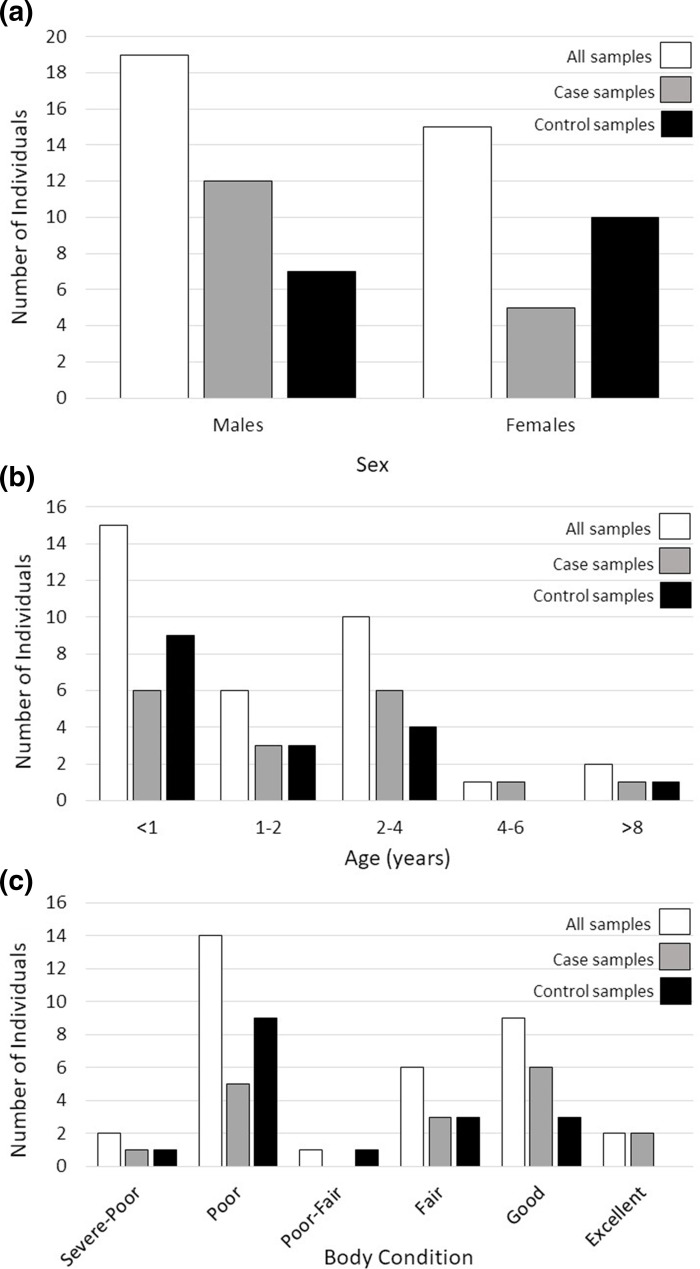
Distribution of sex (**a**), age (**b**) and body condition (**c**) of white-tailed deer (*Odocoileus virginianus*) samples used to identify microbes associated with fatal pneumonia in Wisconsin, USA. Colours correspond to total sample sizes (white bars), subsequently broken down into case (grey bars), and control (black bars) sample groups.

We found no significant differences in the distribution of sex (*P*=0.17), age (*P*=0.57), or body condition (*P*=0.40) variables across our case and control sample groups (Fig. 1), eliminating these variables as potential sources of variation explaining disease outcome in this dataset.

## Discussion

This study presents a preliminary list of microbes identified from DNA extractions that may play a role in the recently observed cases of fatal pneumonia in Wisconsin white-tailed deer. Interestingly, we only identified bacterial species, suggesting that DNA viruses and fungi do not play a critical role in these cases. These results, however, may not be surprising considering that we avoided cases of interstitial pneumonia, which are more likely to involve viral infections. Comparison of the bacterial species identified in our case versus control samples did not reveal any clear candidates as the sole etiologic agent of pneumonia in deer. While this may be the result of the small sample sizes used in this study, it also likely reflects the complex and often polymicrobial nature of pneumonia in both wild and domestic species (e.g. [33,45, 46]). For example, although *M. ovipneumoniae* is thought to be the predominant pathogen responsible for fatal pneumonia of bighorn sheep, the complete aetiology is complex, putatively comprising polymicrobial interactions among several bacteria (e.g. *P. multocida,* leukotoxin positive *Mannheimia haemolytica* [45,47]). In addition, aetiological investigations may be complicated by the effects of disease progression on microbial communities; for example, *M. ovipneumoniae* was the primary pathogen identified in early cases of pneumonia of bighorn sheep, prior to colonization of secondary bacterial pathogens during the chronic disease persistence phase [47,49]. The important role that co-infection may play in facilitating fatal pneumonia in white-tailed deer is supported by our identification of a larger number of putatively pathogenic bacterial species in individuals that died of pneumonia (cases; average=3.5 species/individual), in comparison to those that did not (controls; average=1.8 species/individual).

Importantly, some species identified (e.g. *Escherichia coli*, *Clostridium botulinum*) may be artefacts of field sampling (e.g. contamination or post-mortem bacterial overgrowth). Further, we cannot ignore the potential role that RNA viruses may play in this system. We were unable to screen for RNA viruses as our samples were not preserved to maintain RNA, thus future research efforts could investigate whether RNA viruses play a role in this system.

Among the bacterial species we identified, *Clostridium novyi* was the only putative pathogen that substantially differed (*P*=0.06) between the case and control sample groups, appearing in eight individuals that died of pneumonia (~47 % of case samples), and two that did not (~12 % of control samples). *Clostridium novyi* is a Gram-positive bacterium with several pathogenic strains that have been attributed to diseases in ruminants. For example, bighead disease causes death in rams of domestic sheep (*Ovis aries*) following the invasion of fighting-induced damaged tissues by toxigenic *C. novyi* type A [50]. Additionally, infectious necrotic hepatitis has been attributed to *C. novyi* type B infection in sheep, cattle, goats, and horses [51]. Importantly, although pathogenic *C. novyi* has been associated with diseases of ruminants, its presence in our case samples may alternatively be the result of, rather than the cause of, pneumonia. As an obligate anaerobe, multiplication of *C. novyi* may result as a side-effect of diseases that reduce oxygen in the blood and organs, which may occur if severe lung inflammation results in difficulty breathing. Alternatively, as this bacterium has been associated with tissue decomposition [52], its presence cannot be ruled out as post-mortem bacterial overgrowth.

Although not significantly different between our case and control groups, we did detect some bacterial species that represent pathogens commonly associated with diseases of ruminants, including *M. ovipneumoniae*, *T. pyogenes*, *P. multocida*, *A. phagocytophilum*, and *F. necrophorum. Trueperella pyogenes* and *P. multocida* are opportunistic pathogens that can result in diverse clinical manifestations, including pneumonia [53,54]. In North American white-tailed deer, *T. pyogenes* and *F. necrophorum* have been specifically associated with pneumonia fatalities [55,57]. We also detected *M. ovipneumoniae,* which has been strongly associated with lethal pneumonia in bighorn sheep populations [36,37, 47]. However, while *M. ovipneumoniae* was associated with a single case of pneumonia in Alaskan caribou (*Rangifer tarandus granti*), it has since been shown to be geographically and temporally widespread in populations across Alaska, with no clear association to disease [58]. Lastly, *A. phagocytophilum* is a known cause of tick-borne fever in domestic ruminants [59]; however, infection of white-tailed deer does not appear to result in clinical disease [60,61].

Using aerobic cultures, we confirmed the presence of both *T. pyogenes* and *P. multocida* in our samples, although the varying rates of agreement between the two techniques suggested that some species (i.e. *T. pyogenes*: 80 % agreement between methods) may be more easily cultured than others (i.e. *P. multocida*: identified by culturing in only 20 % of samples with metagenomic sequencing detections; Table S3). Importantly, metagenomic sequencing has the added benefit of identifying species that cannot be detected by culture methods, particularly viruses. In two cases, cultures identified bacterial species not represented in the metagenomic dataset for that individual, suggesting that metagenomic sequencing may also miss some microbes, either at the stage of sequencing (i.e. rare microbes missed during sequencing), or bioinformatic processing (i.e. sequencing errors or database composition that fail to produce sequence matches; Table S3). Given the discrepancies reported here and elsewhere (e.g. [62]), a combined approach will provide the most conservative estimate of microbial diversity until improvements in pathogen discovery are achieved.

A key limitation of the results presented here is the constantly evolving nature of microbial taxonomy. Specifically, the results of our blast searches are reliant on the taxonomic names provided to sequence data at the time it is deposited into a database. Given that genus- and species-level taxonomy is constantly evolving, especially for microbes, it is probable that sequencing data belonging to a single species can exist under multiple names. This has implications for downstream analysis, where, without detailed knowledge of past and present microbial taxonomy, blast detections of a single species occurring under different names may not be recognized or appropriately combined and accounted for. Indeed, in our short list of 20 bacterial species (Table 3), two entries represent the same species; *Paeniclostridium sordellii* is the revised nomenclature for what was once called *Clostridium sordellii* ([63]; combining the two names still does not result in a significant difference between cases and controls). Given the substantial size of metagenomic datasets, it is not feasible to manually check and correct species names to ensure that all detections of each species are grouped across all possible present and former names. Here, if we assume that a species causing an active infection is ultimately fatal, we should be able to detect it at the threshold of 100 sequence matches under at least one of its names; however, we recognize that this threshold may not be met if sequence matches are divided under multiple different names belonging to the same species.

An additional consideration for this work is a lack of power, due to both limited sample sizes and variation in biological variables across our case and control sample groups (Table 1, Fig. 1), which prohibits definitive identification of pathogens associated with pneumonia in deer. Further, although contaminating species resulting from post-mortem overgrowth may be distinguishable via unbiased distribution in case and control sample cohorts, opportunistic infections associated with pneumonia can complicate the identification of causative agent(s). We also acknowledge that sampling an animal during the chronic stage of disease could limit our ability to identify causative agents present during early pathogen colonization and disease progression. For future study, the use of true controls (i.e. fresh tissue sampled from vehicle collisions, capture-associated mortalities or hunter-harvested animals), including infected animals sampled during earlier disease stages, and larger sample sizes may alleviate some of these biases, and statistical modelling can explicitly account for demographic variation (i.e. sex, age, and physical condition). Further, incorporating sampling of lung tissue during regularly conducted field necropsies, where pneumonia cases can be identified via gross lesions, would be a useful tool to facilitate population disease surveillance on a broader geographic scale.

Although we have not yet identified the microorganisms responsible for pneumonia-related fatalities in Wisconsin white-tailed deer, the use of metagenomic sequencing has proven a useful method to identify candidates of interest for further study. Importantly, metagenomic sequencing serves as an unbiased first step to identify putative pathogens across a broad taxonomic spectrum, including fungi, protists, bacteria, and viruses. Coupled with the accessibility of increasingly affordable and portable sequencers, these techniques will continue to improve our ability to identify the microorganisms underlying novel disease outbreaks, with the potential for real-time applications in field settings [64]. Once candidate pathogens have been identified, more targeted sequencing approaches can be used to screen larger numbers of individuals (i.e. 16S/18S amplicon sequencing).

However promising, further method development could improve metagenomic techniques for pathogen identification. For example, metagenomic sequencing datasets of host samples will be predominantly comprised of host DNA sequences, limiting the ability to detect and identify microbial DNA within samples. In this study, we restricted our multiplexing to three samples per flow cell to ensure sufficient sequencing depth to identify all microbes within each sample. However, even with this conservative sequencing approach, the proportion of ‘useful’ data (i.e. sequences not aligning to the host reference genome) was low, ranging from only 0.26–5.59 % of our full datasets. Novel advancements of the MinION sequencing platform aim to address this limitation by implementing an adaptive sampling approach, whereby sequences are compared in real-time to a database, the contents of which (specified by the user) are leveraged to either deplete or enrich sequences from particular taxa [65]. For example, in our case, the draft white-tailed deer reference genome can be used to deplete the sequencing of host DNA, thereby enriching all other species DNA within the sample, including microbes of interest. In addition to facilitating greater sequencing effort for the species of interest, this advancement in sequencing technology will also enable further reductions in the cost of sequencing, as it facilitates greater multiplexing power.

Further reducing the size of our metagenomic sequence datasets was the high proportion of non-host DNA sequences that did not produce blast matches to the NCBI nucleotide database. Here, between 22.76–95.92 % of our queried sequences remained uncharacterized. While a proportion of these unmapped sequences may be attributed to the higher error rates of long-read sequencing platforms [66], this result also highlights the important limitation that we can only detect species that are represented in online genomic databases and leaves a possibility that we have not detected novel pathogen species that may be involved in pneumonia disease of deer. Future efforts will be greatly aided by sequencing projects like the 100K Pathogen Genome Project [67], which aims to sequence the genomes of diverse pathogens across the globe.

As we increasingly rely on genomic resources for the identification and surveillance of pathogens, we must also continue to employ traditional and complementary pathogenesis research methods (e.g. the isolation and culture of microorganisms and reproduction of disease by experimental challenges to address Koch’s postulates) to confirm findings and identify the mechanistic links between pathogens and the diseases they cause [16]. Fully and accurately describing the aetiology of a disease may be a particularly difficult challenge for polymicrobial diseases like pneumonia, as genomic data cannot provide information on the pathogenicity of the microorganisms identified or the infection dynamics of multiple colonizing pathogens that result in host fatality. Indeed, in this study, the presence of many bacterial species in both case and control samples suggests that deer may be carriers of some of these bacteria, which can become opportunistically pathogenic under unknown environmental and/or physiological conditions. For example, the interaction of the normally commensal bacteria *P. multocida* type B with abnormally high humidity and temperature has been attributed to several mass mortality events in saiga antelope (*Saiga tatarica* [68]). Ultimately, although disentangling the relative roles of host immunity, pathogen virulence, and the environment will require combined effort across multiple fields, we demonstrate how metagenomic sequencing can serve as an important first step in identifying putative microorganisms associated with emerging diseases.

## supplementary material

10.1099/mgen.0.001214 Uncited Table S1.
